# First Insights into the Formation of Metal Soaps in Alkyd-Based Paints: A Proof-of-Concept Investigation Using FTIR Spectroscopy

**DOI:** 10.3390/molecules29245840

**Published:** 2024-12-11

**Authors:** Tommaso Poli, Michael P. Haaf, Anna Piccirillo, Abby P. Costa, Rebecca L. Craig, Federica Pozzi

**Affiliations:** 1 Dipartimento di Chimica, Università di Torino, Via Giuria 7, 10125 Turin, Italy; 2 Department of Chemistry, Ithaca College, 953 Danby Road, Ithaca, NY 14850, USA; mhaaf@ithaca.edu (M.P.H.); acosta2@ithaca.edu (A.P.C.); rcraig2@ithaca.edu (R.L.C.); 3 Centro per la Conservazione ed il Restauro dei Beni Culturali “La Venaria Reale”, Via XX Settembre 18, 10078 Venaria Reale, Turin, Italy; federica.pozzi@ccrvenaria.it

**Keywords:** alkyd paints, metal soaps, cultural heritage

## Abstract

The formation of metal soaps or carboxylates in oil paintings is a widely studied topic. Expanding upon the extant research on the subject, this work investigates the formation of metal soaps as pertaining to alkyd-based media. Especially popular in modern and contemporary art, alkyd paints are complex commercial formulations mainly containing a polyester backbone obtained by the condensation of glycerol and phthalic acids, where the third free alcoholic function is esterified with a blend of saturated and unsaturated fatty acids. The formulation may also contain cross-linking agents, dries, and catalysts. Compared to drying oils, alkyd systems have different stability and mobility, likely prompting different kinetics in the formation of metal soaps. This work explores the formation of metal carboxylates in mock-up paints prepared as mixtures of an alkyd binder with potassium hydroxide and three commonly used pigments (smalt, zinc white, and lead white) at ambient conditions and upon artificial aging. As a second step, samples from two contemporary works by Italian artists Franco Garelli and Luciano Minguzzi were investigated for comparison. The results confirm the formation of metal soaps in both mock-up and real paints, paving the way for future research, with significant implications especially for the conservation of modern and contemporary artworks.

## 1. Introduction

The formation of metal soaps, or carboxylates, in oil paintings is a well-known issue that, in the past few decades, has been studied thoroughly by numerous research groups worldwide [1,2]. In modern paints, metal soaps such as zinc or aluminum stearates might be added deliberately to commercial formulations as plasticizers, favoring the dispersion of pigments within the organic medium [3,4]. However, these compounds can also form from the reaction of free fatty acids with cations from pigments or additives as a result of paint aging and degradation [5]. Indeed, oil paint films typically undergo oxidative processes that promote triglyceride cross-linking on the double bonds of unsaturated fatty acids, enabling oil drying [6,7,8]. Other possible degradation phenomena include, for instance, the hydrolysis of the molecules’ ester bond [9]. The presence of humidity mostly in the form of water, impurities, and pollutants within the paint layers might favor the release of acidic functions both from the free saturated fatty acids and their cross-linked counterparts. These free acidic functions are thus available to react with pigment cations, yielding the formation of free carboxylates, or metal soaps, along with more complex ionomeric structures [2,10,11]. The presence of metal soaps in a paint film does not constitute an issue per se, as demonstrated by the fact that most commercial oil paint formulations contain zinc or aluminum stearates as dispersive agents to prevent the separation of the pigment/medium system during the paint’s shelf life. Nevertheless, it has been assessed that the newly formed carboxylate products can migrate, aggregate, and form protrusions within the paint layers, thus compromising their physical and mechanical properties while also altering the surface’s aesthetical appearance [1,12,13,14]. The most common pigments involved in these types of reactions are zinc white, lead white, smalt, and copper-based pigments (the latter are involved to a more limited extent due to their lower reactivity) [1,2,15]. The reactivity of metal soaps typically leads to even more complex interactions at the interface between the paint layers and the uppermost varnish or finishing coating, involving ion exchange processes with terpenic acids from natural resins such as colophony, sandarac, dammar, and copal [16,17,18].

**Figure 1:**
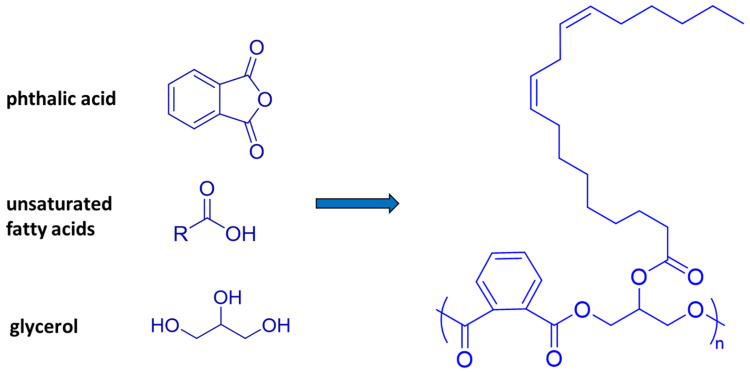
Main components and structure of a generic alkyd resin, where R is typically an unsaturated C16-18 fatty acid.

Building on the extant research on oil paints, this work investigates the formation of metal soaps as pertaining to alkyd-based media. Widely used by world-renowned modern and contemporary artists such as Picasso, Rauschenberg, and Pollock [19,20,21,22], alkyd paints consist of complex commercial formulations mainly containing a polyester backbone obtained by the condensation of 1,2,3-propanetriol (glycerol) and phthalic acids (typically orthophthalic acid), where the third free alcoholic function is usually esterified with a blend of C16-18 saturated and unsaturated fatty acids depending on the exact formulation (Figure 1) [23,24,25,26,27,28].

Commercial formulations may significantly vary and contain different additives (e.g., nitrocellulose), modifiers (e.g., styrene, divinylbenzene), cross-linking agents (e.g., pentaerythritol, sorbitol, maleic acid), and driers or catalysts (often cobalt carboxylates) [23,24,25]. Alkyd-based paints can also be modified with urea/formaldehyde, phenol/formaldehyde, and polyurethane resins to obtain enamels or metal coatings [26,27]. The fatty acid content typically ranges from <40% (short oil) to 70% *w*/*w* (long oil) [27,28]: as a result, a generic alkyd paint formulation may contain on average from 30 to 70% *w*/*w* of fatty acids condensed on the glycerol in mid-position. Based on these features, it is reasonable to hypothesize that alkyd-based paints may undergo similar hydrolysis processes as those described for drying oil, leading to the formation of metal soaps. Compared to drying oil, however, alkyd systems are characterized by different stability and mobility, likely prompting different kinetics in the formation of metal soaps. While the formation of metal soaps in oil paintings and its implications for the conservation of artworks has been the subject of extensive research, only one article to date has focused on alkyd-based paints to the best of our knowledge [29]. The author of that study reported the loss of gloss in zinc white-containing alkyd gloss paints, which was attributed to the presence of protuberances resulting from the formation of zinc soaps, with the hazing effect being worst in the case of alkyd resins with high saturated and oleic acid ester content.

In this context, the present work aims to fill the knowledge gaps on the formation of metal carboxylates in paints that contain alkyd media under controlled aging conditions. In the first phase of this project, a series of mock-up paints were prepared in the laboratory by mixing three commonly used, oil-reactive pigments―namely smalt (a cobalt-colored potassium glass), zinc white (zinc oxide, ZnO), and lead white (basic lead carbonate, Pb(CO)_3_·Pb(OH)_2_)―with an alkyd binder. The selection of materials was based on the available data on pigments that had previously shown high reactivity with oil media, as well as on the frequent occurrence of their cations in modern commercial formulations and substrates: for instance, lead oxides and zinc stearate are typically found as anticorrosive and dispersive agents [3,4,5,30], while zinc is used for galvanized steel. Additionally, zinc oxide is often present in titanium white formulations [31]. Potassium hydroxide (KOH) was also included in the experimentation as a reference since it is a strong base, able to promote the hydrolysis of the polyester backbone fatty acids and is prone to reacting with the newly formed acidic functions. Moreover, potassium is also relevant to the reaction with smalt, as it is the cation involved in the formation of carboxylates. The possibility of metal soap formation in these mock-up paints at ambient conditions and upon artificial aging was assessed by means of transmission FTIR spectroscopy, by detecting the appearance and growth of the -COO-Metal carbonyl band within the same spectral region relevant to drying oils (approximately 1520–1590 cm^−1^) [1,2,4]. The systematic study of mock-up paints was followed by the investigation of samples from two contemporary artworks, namely *Costruzione* (1970) by Franco Garelli and *Uomini* (1971) by Luciano Minguzzi, which both underwent scientific analysis and conservation treatment at the Centro per la Conservazione ed il Restauro dei Beni Culturali (CCR) “La Venaria Reale”, just outside of Turin, Italy. The results of this proof-of-concept work confirmed that the formation of metal soaps occurred both in mock-up and real paints in all cases examined.

## 2. Results

As a preliminary step, FTIR analysis was conducted on the bare alkyd binder reference to confirm the absence of any possible carboxylates (e.g., those containing cobalt, aluminum, and zinc cations), which are often added to the formulation either as driers, catalysts, or dispersive agents [3,5,32]. The results showed that the selected reagent did not contain any unwanted or interfering substances. Section 2.1 and Section 2.2, below, illustrate the main results obtained from a systematic study of the alkyd-containing mock-up paints and from the comparative analysis of two works by Italian artists Garelli and Minguzzi.

### 2.1. Alkyd-Containing Mock-Up Paints

The next four paragraphs discuss data obtained from the FTIR investigation of alkyd-containing potassium hydroxide, smalt, zinc white, and lead white mock-up paints. The selection of spectra shown in the following sections, demonstrating the formation of metal soaps, was collected from mock-up paints prepared with a 1:1 *w*/*w* binder/pigment ratio. Carboxylate formation was also verified for paints with a 2:1 *w*/*w* binder/pigment ratio.

#### 2.1.1. Potassium Hydroxide Mock-Up

**Figure 2:**
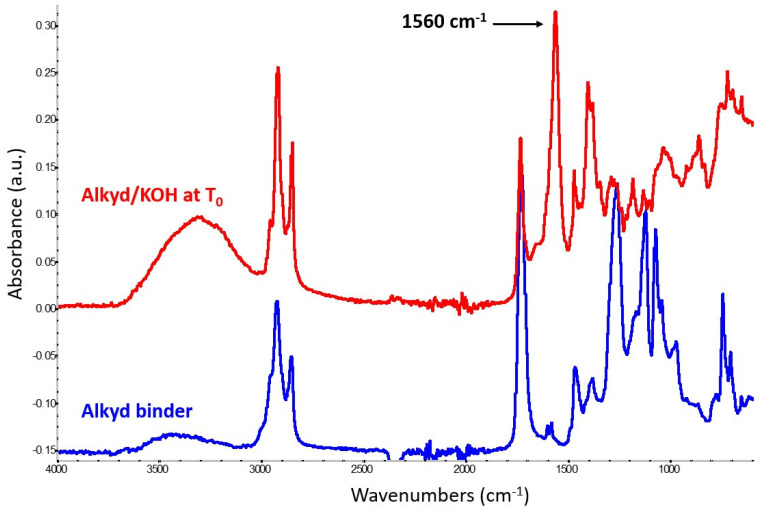
A reference spectrum of the bare alkyd binder (blue trace) compared with the spectrum obtained from the alkyd-containing potassium hydroxide mock-up paint at T_0_ (red trace). The latter shows the formation of potassium carboxylates, identified by a strong band at 1560 cm^−1^.

Analysis of the potassium hydroxide mock-up highlighted the formation of potassium carboxylates, identified by a strong band at 1560 cm^−1^ (Figure 2), which is consistent with data from potassium-containing drying oil paints.

The reaction takes place instantaneously, as the above-mentioned distinctive band was detected immediately after paint mixing. The obtained spectrum also shows a decrease in the C=O ester signal at 1736 cm^−1^, along with a significant decrease in the alkyd backbone bands in the 1000–1300 cm^−1^ region. This observation likely indicates that the potassium hydroxide started to promote the hydrolysis of the polyester chain, resulting in the formation of an OH function, characterized by a broad band centered at 3300 cm^−1^ and other oligomeric oxidated structures.

#### 2.1.2. Smalt Mock-Up Paint

**Figure 3:**
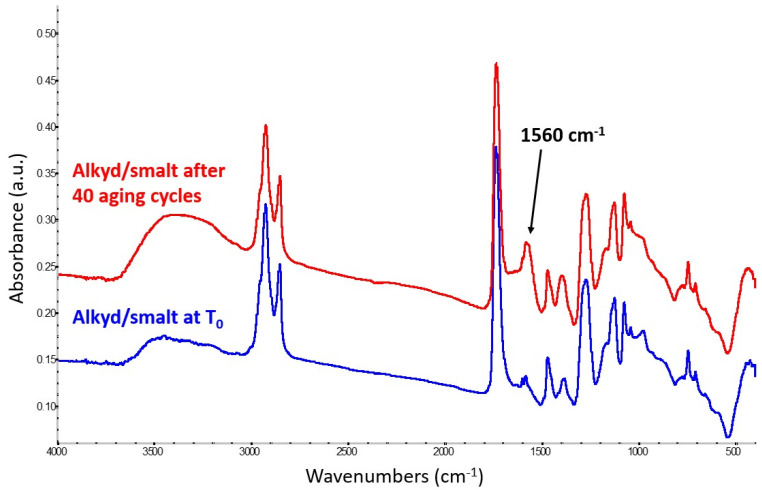
Spectrum of the alkyd-containing smalt mock-up paint at T_0_ (blue trace) compared with the spectrum obtained from the same mixture after 40 aging cycles (red trace). The latter shows the formation of potassium carboxylates, identified by a strong band at 1560 cm^−1^.

As expected, the reaction of the alkyd medium with smalt is slower compared to potassium hydroxide, yet the appearance of a band at 1560 cm^−1^ (Figure 3), related to the formation of potassium carboxylates as in the previous case, is clearly noticeable after 40 cycles of artificial aging.

This suggests that the alkyd medium might interact with smalt in a similar way as drying oil. Potassium is used as a finer and melter in the preparation of smalt. In this case, there are no signs of other ongoing degradation patterns, such as a decrease in the carbonyl band or the appearance of new infrared signals. It is worth pointing out that, nine months after mock-up paint preparation, the band assigned to metal soap formation was also detected in the mock-up paints that were not subjected to artificial aging but rather were kept in the laboratory at ambient conditions.

#### 2.1.3. Zinc White Mock-Up Paint

**Figure 4:**
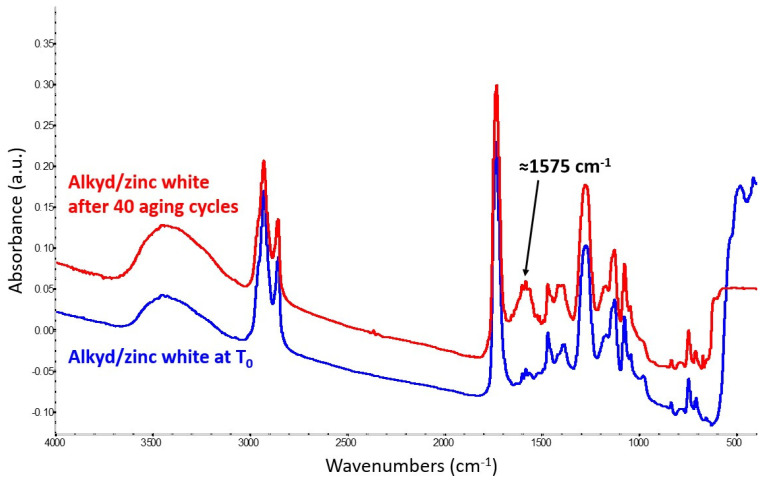
Spectrum of the alkyd-containing zinc white mock-up paint at T_0_ (blue trace) compared with the spectrum obtained from the same mixture after 40 aging cycles (red trace). The latter shows the formation of zinc carboxylates, identified by a strong band at ≈1575 cm^−1^.

A carboxylate-related band, centered at 1575 cm^−1^, was detected in the FTIR spectra obtained from the zinc white mock-up paint, too (Figure 4). In particular, the signal became clearly detectable after 40 aging cycles, around the same wavenumbers as those of zinc soaps from drying oil paints.

This band is broader than the one originating from potassium hydroxide likely due to the simultaneous presence of mono- and di-carboxylated zinc salts in the sample analyzed and to an overall greater structural inhomogeneity. As in the case of smalt, nine months after mock-up paint preparation, an FTIR band related to metal soap formation was also noticed in the mock-up paints kept at ambient conditions, while the spectra show no evidence of the degradation of the alkyd structure.

#### 2.1.4. Lead White Mock-Up Paint

**Figure 5:**
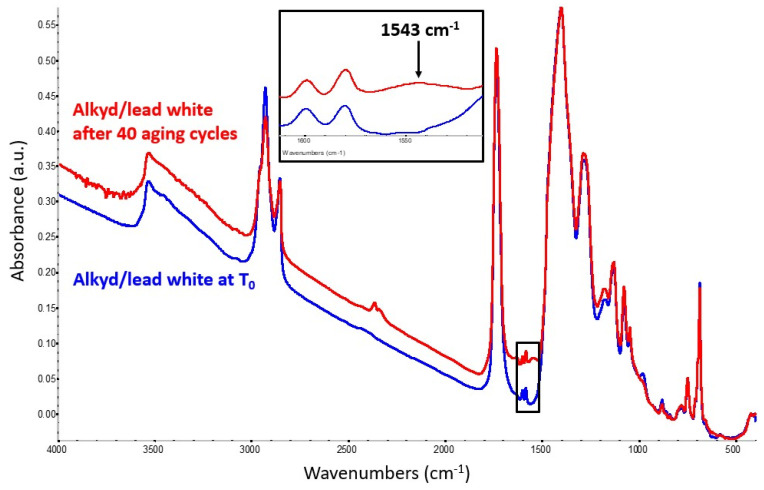
Spectrum of the alkyd-containing lead white mock-up paint at T_0_ (blue trace) compared with the spectrum obtained from the same mixture after 40 aging cycles (red trace). The latter shows the formation of lead carboxylates, identified by a weak band at 1543 cm^−1^.

In the alkyd/lead white mock-up paint, the formation of lead carboxylates was barely noticeable even after 40 aging cycles (Figure 5), though the band’s observed location (1543 cm^−1^) corresponds exactly to that detected for lead soaps originating from lead-containing drying oil paints. It is not clear if such a slow reaction might be due to the actual reactivity of lead ions with the alkyd medium or to the type of lead white used in our experiment, in which the relative proportion of cerussite (Pb(CO)_3_) and hydrocerussite (Pb(CO)_3_·Pb(OH)_2_) is unknown. The ratio between these two components may vary significantly in lead white paint formulations. The spectra collected show no signals of decay in the alkyd structure and, in this case, metal soap formation was not detected in the mock-up paints kept at ambient conditions even after nine months of aging.

### 2.2. Contemporary Artworks

After carrying out systematic work on the mock-up paints, confirming the occurrence of metal soaps in the presence of alkyd binders, FTIR data from previous studies conducted on two contemporary artifacts featuring alkyd-based paints were reviewed to assess the possible presence of carboxylates. The main results are shown below.

#### 2.2.1. Franco Garelli, *Costruzione* (1970)

**Figure 6:**
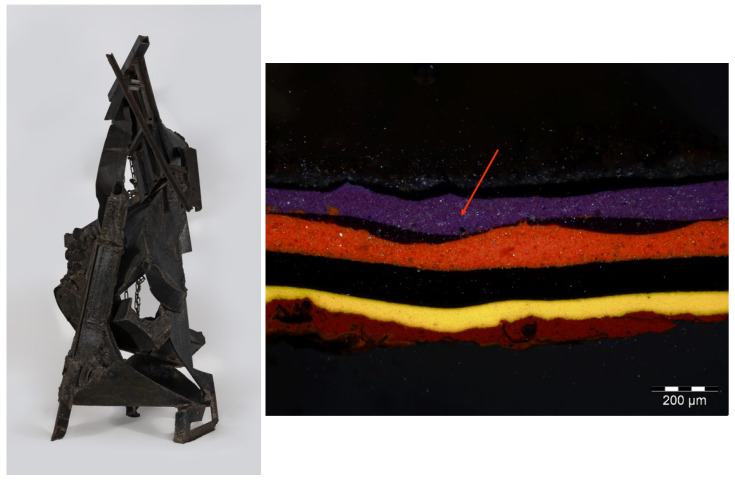
(**Left**) Franco Garelli, *Costruzione* (1970). (**Right**) Visible light image of a cross section removed from the painted decoration of a side metal bar. The arrow indicates a violet paint layer that contains zinc white and Prussian blue.

An exponent of the Informal movement, Franco Garelli (1909–1973) was an Italian physician, painter, sculptor, and ceramist who was presented to the CoBra group by Asger Jorn and later followed the Japanese Gutai experience. *Costruzione* (1970) is representative of the artistic and cultural history of the city of Torre Pellice, Turin, and is one of the most relevant artifacts featured in the Filippo Scroppo Civic Gallery (Figure 6). During conservation treatment at the CCR “La Venaria Reale”, a few multi-layered samples were removed from the work and mounted as cross sections for micro-invasive analysis to gain insight into the materials and techniques used. Microscopic observation of a sample collected from the painted decoration of a side metal bar highlighted a sequence of layers of different colors, consisting of mixtures of pigments with binders of various nature (acrylic, vinylic, and alkyd-based). This complex stratigraphy might be related to deliberate compositional changes on the artist’s part and/or to the reuse of elements from other artifacts.

**Figure 7:**
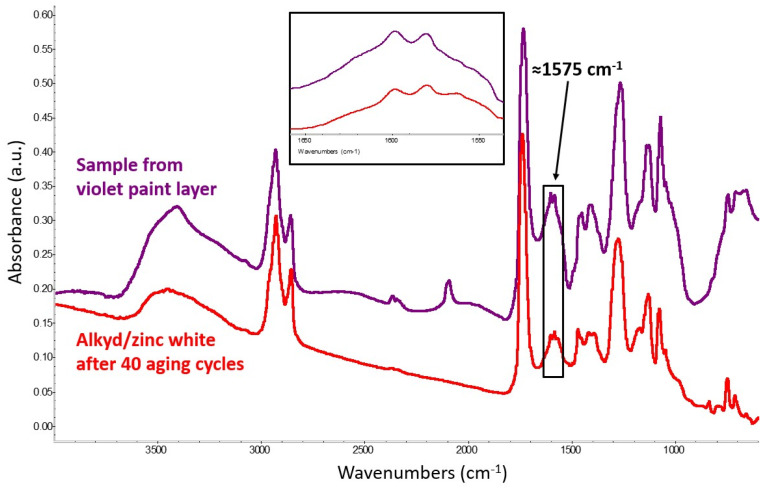
Spectrum of a violet layer in Garelli’s work (violet trace) compared with the spectrum obtained from the alkyd-containing zinc white mock-up paint after 40 aging cycles (red trace). Both show the formation of zinc carboxylates, identified by a strong band at ≈1575 cm^−1^.

Investigations by means of scanning electron microscopy with energy-dispersive X-ray spectroscopy (SEM/EDS) showed that the violet layer, indicated by an arrow in Figure 6, consists of a mixture of zinc white and Prussian blue. Transmission FTIR analysis of a loose scraping from the same layer confirmed the presence of Prussian blue with an alkyd binder. Aside from the pigment’s characteristic C≡N stretching at 2091 cm^−1^, spectra from this material, all featuring a strong band at 1575 cm^−1^ attributed to the formation of zinc carboxylates, show a close correspondence in terms of overall shape and wavenumbers with data acquired from the alkyd-containing zinc white mock-up paint that was prepared in the laboratory and artificially aged (Figure 7).

**Figure 8:**
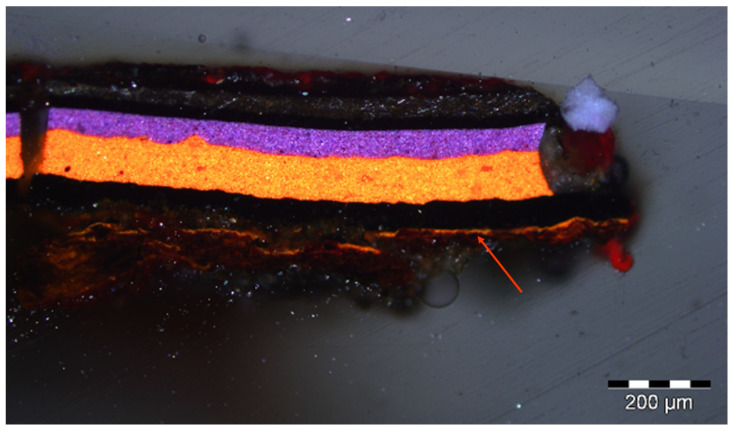
Visible light image of a cross section removed from a fastening element near the chain on Garelli’s work. The arrow indicates a thin, orange layer of minium applied directly onto the metal, below the paint layers, likely serving an anticorrosive purpose.

Other cross sections from the same artwork, including a sample removed from a fastening element near the chain, display a thin, orange layer of minium (lead oxide, 2PbO·PbO_2_) applied directly onto the metal, below the paint layers, likely serving an anticorrosive purpose (Figure 8).

**Figure 9:**
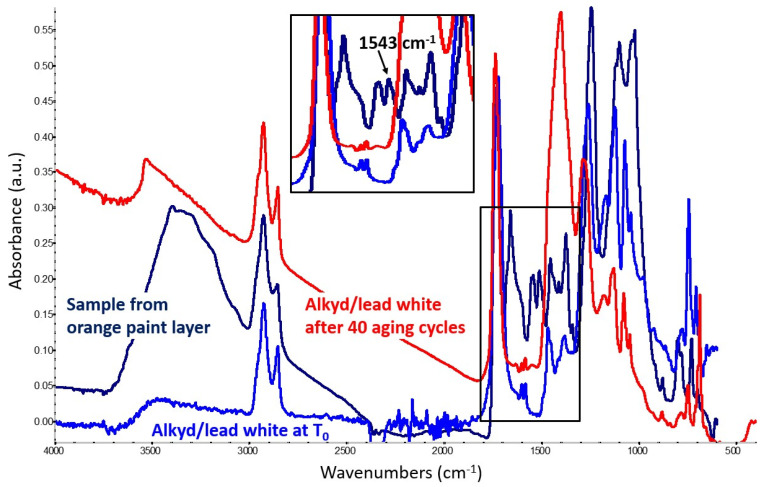
Spectrum of a minium layer sample from Garelli’s work (dark blue trace) compared with spectra obtained from the alkyd-containing lead white mock-up paint at T_0_ (light blue trace) and after 40 aging cycles (red trace). The first and latter show the formation of lead carboxylates, identified by a band at 1543 cm^−1^ that appears stronger in the case of the artwork’s sample.

Transmission FTIR spectra of a loose scraping from this lead-containing layer revealed the presence of an alkyd binder, along with polyvinyl acetate (PVA) likely from an adjacent layer (with main signals at 1244 and 1026 cm^−1^). The 1543 cm^−1^ band, ascribable to the formation of lead carboxylates, appears significantly stronger in this artwork’s sample compared to the aged alkyd/lead white mock-up paint (Figure 9). It is worth noticing that, albeit forming the same type of carboxylate, the lead cations involved in the reaction in this case do not originate from the lead white pigment, but from lead oxide, possibly leading to different reactivity.

#### 2.2.2. Luciano Minguzzi, *Uomini* (1971)

**Figure 10:**
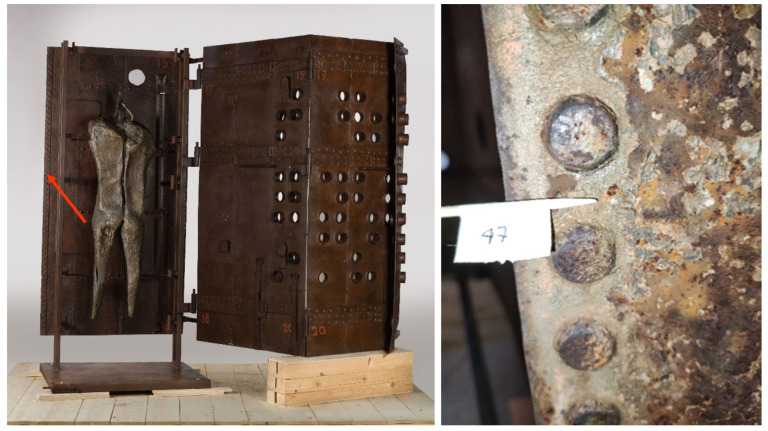
(**Left**) Luciano Minguzzi, *Uomini* (1971). (**Right**) Detail of the sampling site. The arrow indicates the exact location subjected to sampling.

Luciano Minguzzi (1911–2004) was an Italian sculptor, medallist, and engraver who was selected to design the fifth door of the Milan Cathedral and is currently featured at the Peggy Guggenheim collection in Venice. *Uomini* (1971) is a large-scale installation made of metal alloys and resins, normally located in Saint-Vincent, Aosta Valley, which recently underwent conservation treatment at the CCR “La Venaria Reale” (Figure 10).

**Figure 11:**
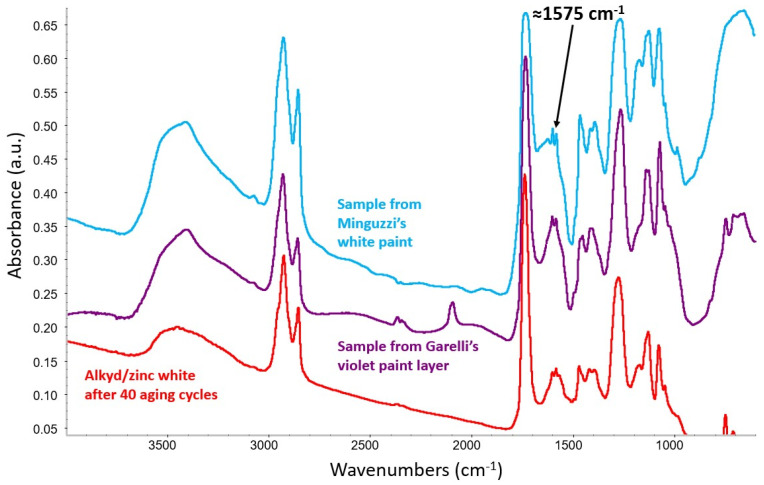
Spectrum of a white paint sample from Minguzzi’s work (light blue trace) compared with spectra obtained from Garelli’s violet layer (violet trace) and the alkyd-containing zinc white mock-up paint after 40 aging cycles (red trace). All spectra show the formation of zinc carboxylates, identified by a strong band at ≈1575 cm^−1^.

After non-invasive XRF investigations of selected spots of interest on the artwork’s surface, a sample of degraded white paint removed from the proper right edge of one of the panels was characterized with transmission FTIR spectroscopy. The analysis highlighted the presence of zinc white dispersed in an alkyd resin. As in the case of Garelli’s violet paint layer, the FTIR spectrum shows a distinctive band centered at 1575 cm^−1^, ascribable to the formation of zinc carboxylates (Figure 11).

## 3. Discussion

As expected, the systematic work on mock-up paints illustrated herein highlighted a similar reactivity in alkyd-based media to that of drying oils due to an abundance of fatty acids in their formulations (with a theoretical minimum of 30% *w*/*w*). In the FTIR spectra, the metal carboxylates originating from the reaction of an alkyd binder with the chosen pigments gave rise to absorption bands at the same wavenumbers as metal soaps from drying oil. The choice of cations in this work aimed to verify the formation of metal soaps in alkyd-based paints under controlled aging conditions, starting from pigments that are known to be the most reactive in drying oil media. While smalt and lead white are not present in modern alkyd-based paint formulations, their cations are commonly available in other materials that are relevant to cultural heritage research: for instance, potassium can be found in stones, plasters, and mortars employed in wall painting, while lead compounds are often used as anticorrosive layers, as also reported in the Garelli case study.

At this stage, a direct comparison of the kinetics of metal soap formation is challenging because the reactivity of each material strongly depends on the cation’s availability, i.e., on the compound in which it is found and its chemical environment. On a qualitative level, based on a preliminary comparison of the relative intensities of the observed carboxylate bands, we detected a greater reactivity for zinc white compared to smalt, and a greater reactivity of both these pigments compared to lead white. However, it must be taken into consideration that the seemingly lower reactivity of lead might also depend on the availability of the lead cations within the specific material used, in which the exact hydrocerussite/cerussite ratio is unknown. Additionally, materials containing the same cation, such as lead white and minium, might prompt different scenarios, even though the formation of carboxylates was verified at the same wavenumbers: in other words, the formation of lead carboxylates gives rise to a band at 1543 cm^−1^ when lead cations originate from both a lead white pigment and from an anticorrosive layer of minium.

Compared to drying oil, the kinetics of metal soap formation in alkyd-based paints are expected to be slower, likely due to the lower number of fatty acids present and to the overall lower mobility of the system under study, in which fatty acids are not attached to glycerol to form triglycerides but to a polymeric polyester backbone.

Scientific analysis revealed an exact correspondence between metal carboxylates found in laboratory mock-up paints and samples from actual artifacts. This confirms that the same type of metal carboxylates observed in the mock-up paints also form in real artwork, even though the latter are likely to contain commercial alkyd binders or enamels whose formulations might be more complex and diverse than the media used here.

The inhomogeneity of alkyd-based paints might pose a challenge to the conservation of artworks, as modern and contemporary artists often do not pursue pure alkyd media, but rather make extensive use of lacquer, spray paints, or enamels originally formulated for different purposes (including, but not limited to, metal coating and surface finishing). These products can be heavily modified, for example, by adding melamine–formaldehyde resins, typically yielding significantly more reticulated structures than alkyd paint media. Moreover, the system’s cross-linking rate and “stiffness” might deeply affect the hydrolizability of the fatty acids, their mobility, and their ability to interact with cations. As a matter of fact, no signs of migration or aggregation of the newly formed carboxylates were detected in the mock-up paints or contemporary artwork samples, likely due to their relatively short lifespan so far and possibly to the system’s lower mobility. On the other hand, one must consider that works featuring alkyd-based paints are often significantly more recent than oil paintings, which means that the onset of issues in alkyd-containing artwork might be yet to occur.

## 4. Materials and Methods

### 4.1. Preparation of Mock-Up Paints

As virtually no literature exists on the formation of metal soaps in alkyd-based paints, the choice of pigments for this proof-of-concept study was based on the available data on pigments that had previously shown high reactivity with oil media. With this consideration in mind, mock-up paints were prepared as mixtures of an alkyd binder with potassium hydroxide and three commonly used pigments (smalt, zinc white, and lead white). The alkyd binder, described by the manufacturing company as “Médium Alkyde Qualitè Beaux Arts-Satinè”, was purchased from Lefranc Bourgeois (Paris, France). Smalt, zinc white, and lead white were supplied by Kremer Pigmente (Aichstetten, Germany), while potassium hydroxide was supplied by Merck (Boston, MA, USA).

Two sets of four mock-up paints were prepared with two different binder/pigment ratios (1:1 and 2:1 *w*/*w*). The resulting paints were applied onto silicon float zone polished windows (13 mm diameter × 1 mm thickness) purchased from Crystran Ltd. (Dorset, UK). In addition, a mock-up sample with the bare alkyd resin and no pigments was prepared as a reference.

Each mock-up was prepared in three replicas to ensure experiment reproducibility. Three mock-up replicas for each cation were kept for nine months at ambient conditions without direct exposure to sunlight (22 °C and 55% relative humidity). Three mock-up replicas for each cation were also subjected to artificial aging, undergoing wet (3 days, 22 °C, and 95% relative humidity) and dry (3 days in the oven, 65 °C, and 20% relative humidity) alternated cycles for up to nine months, totaling 40 aging cycles. The initial FTIR measurements were conducted one week after paint film curing; after that, analyses were carried out once a day for the first week and once a week for the rest of the aging timeframe.

### 4.2. FTIR Measurements

FTIR analysis was performed with a Bruker Vertex 70 FTIR spectrometer (Billerica, MA, USA) equipped with a DTGS detector, coupled with a Bruker Hyperion 3000 infrared microscope with a mercury cadmium telluride (MCT) detector. Mock-up paints applied onto silicon float zone polished windows were analyzed as is using the system’s optical bench, while samples from the contemporary works were examined upon compression in a diamond cell through a 15× objective. Data were collected in transmission mode, in the 4000–400 cm^−1^ spectral range, at a spectral resolution of 4 cm^−1^, as the sum of 64 scans.

## 5. Conclusions

While the formation of metal soaps in oil paintings has been the subject of extensive research to date, virtually no data exist in the literature on this phenomenon as pertaining to alkyd-based artists’ paints. In this work, the possibility of metal carboxylate formation in the presence of alkyd media was assessed for the first time through a systematic investigation of mock-up paints by means of transmission FTIR spectroscopy. A series of mock-up paints were prepared by mixing a commercial alkyd binder with potassium hydroxide and three widely used pigments (i.e., smalt, zinc white, and lead white). The resulting paints were applied onto silicon wafers and subjected to natural and artificial aging. Measurements were taken at regular intervals, after curing the paint film, over a period of nine months. After testing the mock-ups, samples from two contemporary installations by Italian artists Franco Garelli and Luciano Minguzzi were also investigated for comparison. The results confirmed the formation of metal carboxylates in both mock-up paints and artwork samples, revealing different reactivity for the materials selected that strongly depends on the cations’ availability within their own chemical environment. Remarkably, the data collected highlight an exact correspondence between metal carboxylates found in laboratory mock-ups and samples from actual artifacts, even though the latter typically feature commercial alkyd binders with more complex formulations than the media used here. Overall, the work presented herein provides readers with preliminary, yet essential insights on a topic of special interest in the field of cultural heritage, paving the way for future research with potentially significant implications, especially from the point of view of the conservation of modern and contemporary artworks.

In the future, this proof-of-concept study involving mock-up paints will be extended to a selection of ready-to-use commercial alkyd-based tube paints, spray paints, and enamels. In addition to evaluating the kinetics of metal carboxylate formation in a series of materials of interest, a thorough investigation of the paint film’s physical and mechanical properties, behavior, and changes thereof will be conducted. Future research on this topic might also include an in-depth characterization of the newly formed carboxylates so as to understand the impact of metal soap formation on the behavior of alkyd-based paints and predict possible conservation issues arising from these degradation processes. At this research stage, it is unclear whether the metal soaps formed within a work’s paint layers in the presence of alkyd binders may undergo migration and/or aggregation as is observed in the case of drying oils: this aspect will be investigated using imaging techniques. A detailed comparison between the kinetics of metal soap formation in oil-based and alkyd-based paints will be also performed.

From an analytical standpoint, FTIR spectroscopy is currently the technique of choice for the detection and characterization of metal carboxylates in paints. This is thanks to the availability of benchtop and portable instrumentation as part of the basic laboratory infrastructure, not only in highly specialized research institutions and universities, but also in most museums and conservation centers. More work needs to be done to identify additional, reasonably accessible instrumental techniques that could be employed along with FTIR in this type of studies: this might be especially useful in the case of complex mixtures, where the distinctive infrared carboxylate-related bands are sometimes obscured or disguised by the overlapping signals of other materials.

## Data Availability

All data are either displayed in this article or available from the corresponding authors upon reasonable request.
